# International Medical Congress

**Published:** 1876-01

**Authors:** 


International Medical Congress.—The medical societies of Philadelphia, animated by a just spirit of patriotism, and an earnest desire to unite with their fellow-citizens in celebrating the centennial birthday of American independence, have taken the initiatory steps for the formation of an International Medical Congress, by the appointment of delegates from their respective bodies, who were empowered to organize and perfect a scheme for the above purpose. In accordance with the authority thus given, the delegation has organized the Centennial Medical Commission, with the following officers:

President, Samuel D. Gross, M.D., LL.D., D.C.L. Oxon.

Vice-Presidents, W. S. W. Ruschenberger, M.D., U. S. N.; Alfred Stille, M.D.

Recording Secretary, William B. Atkinson, M.D.

American Corresponding Secretaries, Dan'l G. Brinton, M.D.; William Goodell, M.D.

Foreign Corresponding Secretaries, Richard J. Dunglison, M.D.; R. M. Bertolet, M.D.

Treasurer, Caspar Wister, M.D.

Arrangements have been made for the holding of the Congress in the city of Philadelphia, to begin on the 4th and to terminate on the 9th of September, 1876. The Commission propose the following general plan for the organization and business of the Congress:

I. The Congress shall consist of delegates, American and foreign; the former representing the American Medical Association and the State and Territorial Medical Societies of the Union; the latter the principal medical societies of other countries.

II. The officers shall consist of a President, ten Vice-Presidents, four Secretaries, a Treasurer, and a Committee of Publication, to be elected by the Congress at its first session, on the report of a Committee of Nomination.

III. The morning sessions of the Congress shall be devoted to general business and the reading of discourses; the afternoons to the meetings of the sections, of which there shall be nine, viz:

1. Medicine, including Pathology, Pathological Anatomy and Therapeutics.

2. Biology, including Anatomy, Histology, Physiology and Microscopy.

3. Surgery.

4. Dermatology and Syphiology.

5. Obstetrics and Diseases of Women and Children.

6. Chemistry, Toxicology and Medical Jurisprudence.

7. Sanitary Science, including Hygiene and Medical Statistics.

8. Ophthalmology and Otology.

9. Mental Diseases.

IV. The language of the Congress shall be English, but not to the exclusion of any other language in which members may be able to express themselves more fluently.

Gentlemen intending to make communications upon scientific subjects will please notify the Commission at the earliest practicable date, in order that places may be assigned them on the programme.

In order to impart to the Congress a thoroughly international character, invitations to send delegates will be extended to all the prominent medical societies in Europe, Mexico, the British Dominions, Central and South America, the Sandwich Islands, the East and West Indies, Australia, China and Japan. Invitations will also be tendered to medical gentlemen of high scientific position; and distinguished visitors may be admitted to membership by a vote of the Congress.

Among the advantages arising from such a convocation as this, not the least important will be the opportunity afforded its members for the interchange of friendly greetings, the formation of new acquaintances, and the renewal and cementing of old friendships.

The Centennial Medical Commission tender in advance to their brethren in all parts of the world a cordial welcome, and a generous hospitality during their sojourn in the "Centennial City."

The Congress will be formally opened at noon, on Monday, the fourth day of September, 1876.

The registration book will be open daily from Thursday, Aug. 31, from 12 to 3 P.M., in the hall of the College of Physicians,

N. E. corner 13th and Locust streets. Credentials must in every case be presented.

Gentlemen attending the Congress can have their correspondence directed to the care of the College of Physicians of Philadelphia, N. E. corner of Locust and Thirteenth streets, Philadelphia, Penn.

There is every reason to believe that there will be ample hotel accommodation for all strangers visiting Philadelphia in 1876. Further information may be obtained by addressing the Corresponding Secretaries.

All communications must be addressed to the appropriate Secretaries.

William B. Atkinson, 1400 Pine street, Philadelphia, Recording Secretary.

Daniel G. Brinton, 2027 Arch street; William Goodell, 20th and Hamilton streets, American Corresponding Secretaries.

Richard J. Dunglison, 814 N. 16th street; R. M. Bertolet, 113 S. Broad street, Foreign Corresponding Secretaries.

EXECUTIVE COMMITTEES FOR THE SOUTHERN STATES.

*Maryland.*—C. M. Ellis, of Elkton; Christopher Johnston, of Baltimore; Nathan R. Smith, of Baltimore.

*Virginia.*—J. L. Cabell, of University of Virginia; L. S. Joynes, of Richmond; Hunter McGuire, of Winchester

*North Carolina.*—P. E. Hines, of Raleigh; W. A. B. Norcom, of Edenton.

*South Carolina.*—E. Geddings, of Charleston; R. W. Gibbes, of Columbia; F. Peyre Porcher, of Charleston.

*Georgia.*—R. D. Arnold, of Savannah; Robert Battey, of Rome; L. A. Dugas, of Augusta.

*Florida.*—F. D. Lente, of Palatka; R. D. Murray, of Key West.

*Alabama.*—William O. Baldwin, of Montgomery; Claudius H. Mastin, of Mobile.

*Mississippi.*—Wirt Johnson, of Jackson; J. M. Taylor, of Corinth.

*Louisiana.*—S. M. Bemis, of New Orleans; S. Dickson Bruns, of New Orleans; T. G. Richardson, of New Orleans.

*Tennessee.*—W. K. Bowling, of Nashville; Paul F. Eve, of Nashville.

*Kentucky.*—R. C. Hewett, of Louisville; J. D. Jackson, of Danville; D. W. Yandell, of Louisville.

